# Supplementary material for: Effect of out-of-school visual art activities on academic performance. The mediating role of socioeconomic status
Source: PLoS One. 2024 May 14;19(5):e0298901. doi: 10.1371/journal.pone.0298901 (PMC11093298; doi:10.1371/journal.pone.0298901)
Supplement: Attachment — Submitted filename: Manuscript ID PONE-D-23-15066.docx [file pone.0298901.s002.docx]

**Manuscript ID:** **PONE-D-23-15066**

**Does Parental time or money spent on children's visual art activities close the academic performance gap? Evidence from Contemporary China**

This paper is about parental time or money spent on children's visual art activities applying a cross-sectional survey study and then analyzing it with a quantitative approach. The topic is relevant and very up-to-date in the Social and Behavioral Sciences field. In addition, the study is also applicable to the mission of the journal in highlighting children’s behavioral studies. However, in my opinion, there are some flaws with this paper, which I address in turn below. I show below some ideas for the author on how to make the paper even more robust.

1. The title needs to be clearer. The word “contemporary” looks odd.
2. The submission has good identification of the problems/issues related to the topic, the topic's importance, and the research gap. Though it is a rigorous study in the context of cross-sectional data, the study has lacking research aim/purpose as well as the research question. What actually author(s) want to answer the question by conducting this research and how this study contributes to the literature and practically – children/parents? What’s new in this study? compared to others?
3. The literature review sounds good. But I am worried about many old unnecessary citations. I strongly suggest to the author(s) reduce the unnecessary citation.
4. Though the methodology looks good, and the author(s) applied random sampling for this study, is there any specific framework for determining sample size? Also, I appreciate the ethical approval number with evidence. I also expect the details of the data collection procedure.
5. The results are supported by the correct data analysis, they are sufficient.
6. Some minor revisions are needed, specifically in the section on implications. The implications are not conclusions significantly. Though the research findings support the results to be classified into practical implications, the theoretical contribution is lackings.
7. The manuscript still not yet representing good academic writing and needs language editing.

In summary, please note that I am only trying to point out that your paper does not satisfy the journal's quality requirements at this stage of development, and further MAJOR Revision is needed —however, exciting topic. I hope my comments will help the authors improve the article. Good luck.
