# Supplementary material for: Effect of out-of-school visual art activities on academic performance. The mediating role of socioeconomic status
Source: PLoS One. 2024 May 14;19(5):e0298901. doi: 10.1371/journal.pone.0298901 (PMC11093298; doi:10.1371/journal.pone.0298901)
Supplement: Attachment — Submitted filename: Point.edited.docx [file pone.0298901.s003.docx]

**Point-by-Point Response to Reviewers’ Comments**

Dear Editors and reviewers,

I am writing this letter on behalf of my coauthor regarding our Manuscript ID: PONE-D-23-15066, entitled " **Effect of out-of-school Visual Art Activities on Academic Performance. The Mediating Role of Socioeconomic Status**." We want to thank the respected editors and reviewers for providing constructive comments and suggestions for the manuscript to be considered for publication with minor adjustments. As you can see below, we have responded to all reviewer's inquiries before the manuscript is published.

**Comments**

**Comment:** Does Parental time or money spent on children's visual art activities close the academic performance gap? Evidence from Contemporary China

**Author Response:** We agree with the respected reviewer’s comment and refined the revised manuscript title.

**Comment:** The submission has good identification of the problems/issues related to the topic, the topic's importance, and the research gap. Though it is a rigorous study in the context of cross-sectional data, the study has lacking research aim/purpose as well as the research question. What actually author(s) want to answer the question by conducting this research and how this study contributes to the literature and practically – children/parents? What’s new in this study? compared to others?

**Author Response:** Please refer to pages 4, 5, and 11, which demonstrate the study's contribution and the research hypothesis.

**Comment:** The literature review sounds good. But I am worried about many old unnecessary citations. I strongly suggest to the author(s) reduce the unnecessary citation.**Authors Response: We kindly want to inform the reviewer that, putting full names would take make the organization of the manuscript out of format, messy and hard to follow.**

**Author Response: It was a great suggestion. Necessary amendments are addressed.**

**Comment:** Though the methodology looks good, and the author(s) applied random sampling for this study, is there any specific framework for determining sample size? Also, I appreciate the ethical approval number with evidence. I also expect the details of the data collection procedure.

**Author Response: The suggestion is addressed.**

**Comment:** Though the research findings support the results to be classified into practical implications, the theoretical contribution is lackings.

**Author Response: Based on the findings of the study. We addressed the theoretical implication of the study, which can be exhibited in the revised manuscript.**

**Comment:** The manuscript still not yet representing good academic writing and needs language editing.

**Author Response: A professional Academic editing service has been used to ensure the language accuracy of the manuscript.**
