# Supplementary material for: Effect of out-of-school visual art activities on academic performance. The mediating role of socioeconomic status
Source: PLoS One. 2024 May 14;19(5):e0298901. doi: 10.1371/journal.pone.0298901 (PMC11093298; doi:10.1371/journal.pone.0298901)
Supplement: Attachment — Submitted filename: Cover Letter.edited (4).edited.docx [file pone.0298901.s004.docx]

**Cover Letter**

September 13, 2023

Dear Editors and reviewers,

I am writing this letter on behalf of my coauthor regarding our manuscript PONE-D-23-15066R1, entitled " **Effect of out-of-school Visual Art Activities on Academic Performance. The Mediating Role of Socioeconomic Status**". We thank the respected editors and reviewers for providing constructive comments and suggestions for the manuscript to be considered for publication with minor adjustments. As you can see below, we strived to attempt every inquiry raised by the three reviewers before the manuscript was ready for publication.

**Reviewer 1 Comments**

**Comment 1:** Abstract should be equipped with used methodology and policy recommendations.

**Author Response:** Thank you for the compliment on the rigorous review the authors did. In addition, per the reviewer's suggestion, we included relevant and evidence-based implications and the methodology employed in the present study.

**Comment 2:** This section fails to explain the concepts of study, novelty and significance of the study.

**Author Response:** The revised introduction section of the manuscript ensured that relevant and recent evidence has been used to strengthen the argument and highlight the particular purpose of the study.

**Comment 3:** The used methodology MANOWA should be explained in this section

**Author Response:** Relevant explanations in the method section provide vivid information for the reader.

**Comment 4:** Conclusion section is not present in the study.

**Author Response:** Thank you for the significant insight, which avoids confusion. The Conclusion section has been addressed to provide a comprehensive reflection of the study.

**Reviewer 2 Comments**

**Comment 1:**  No research questions/objectives are added

**Authors Response:** Thank you for the suggestion. We have made sure that the main purpose of the study is highlighted at the end of the introduction and literature review section, and regarding the research questions, under the present study section, all the research hypotheses or questions are stated. Kindly refer to the revised manuscript.

**Comment 2:** Sample size determination was not added

**Author's Response: Thank you for the concern. W**e provided relevant explanations for that.

**Comment 3:** How is the data of the parents obtained and how is it matched with the corresponding child? For this kind of sensitive information, verbal consent was insufficient. Did the parents know that the child's academic scores will be obtained from the schools and provided to the researchers?

**Author Response:** Verbal consent was a supporting means we used to explain the objective of our study for those parents who wanted to give verbal consent; however, written consent was obtained from the rest. In addition, as we mentioned in the methodology section, the teachers and school principals helped us obtain each sampled student's academic performance, and the teachers requested participants to obtain their parental educational and monthly income with consent.
